# Oncologic outcomes in men with metastasis to the prostatic anterior fat pad lymph nodes: a multi-institution international study

**DOI:** 10.1186/s12894-015-0070-1

**Published:** 2015-08-01

**Authors:** Young Suk Kwon, Yun-Sok Ha, Parth K. Modi, Amirali Salmasi, Jaspreet S. Parihar, Neal Patel, Izak Faiena, Michael May, David I. Lee, Elton Llukani, Tuliao Patrick, Koon Ho Rha, Thomas Ahlering, Douglas Skarecky, Hanjong Ahn, Seung-Kwon Choi, Sejun Park, Seong Soo Jeon, Yen-Chuan Ou, Daniel Eun, Varsha Manucha, David Albala, Ketan Badani, Bertram Yuh, Nora Ruel, Tae-Hwan Kim, Tae Gyun Kwon, Daniel Marchalik, Jonathan Hwang, Wun-Jae Kim, Isaac Yi Kim

**Affiliations:** Section of Urologic Oncology, Rutgers Cancer Institute of New Jersey and Rutgers Robert Wood Johnson Medical School, The State University of New Jersey, 195 Little Albany Street, New Brunswick, NJ USA; Department of Urology, School of Medicine, Kyungpook National University Medical Center, Daegu, Korea; Department of Pathology, Robert Wood Johnson Medical School, New Brunswick, NJ USA; Division of Urology, University of Pennsylvania, Philadelphia, PA USA; Department of Urology, College of Medicine, Yonsei University, Seoul, Korea; Department of Urology, UC Irvine School of Medicine, Orange, CA USA; Department of Urology, Asan Medical Center, University of Ulsan College of Medicine, Seoul, Korea; Department of Urology, University of Ulsan College of Medicine, Ulsan University Hospital, Ulsan, Korea; Department of Urology, Samsung Medical Center, Sungkyunkwan University School of Medicine, Seoul, Korea; Prostate Disease Center, Taichung Veterans General Hospital, Taichung, Taiwan; Department of Urology, Temple University School of Medicine, Philadelphia, PA USA; Department of Pathology, Temple University School of Medicine, Philadelphia, PA USA; Associated Medical Professionals, Syracuse, NY USA; Department of Urology, Icahn School of Medicine at Mount Sinai Hospital, New York, NY USA; Division of Urology and Urologic Oncology, City of Hope National Medical Center, Duarte, CA USA; Department of Urology, Georgetown University, Washington, DC USA; Department of Urology, Chungbuk National University College of Medicine, Cheongju, Korea

**Keywords:** Lymph node metastases, Prostate anterior fat pad, Prostate cancer

## Abstract

**Background:**

The presence of lymph nodes (LN) within the prostatic anterior fat pad (PAFP) has been reported in several recent reports. These PAFP LNs rarely harbor metastatic disease, and the characteristics of patients with PAFP LN metastasis are not well-described in the literature. Our previous study suggested that metastatic disease to the PAFP LN was associated with less severe oncologic outcomes than those that involve the pelvic lymph node (PLN). Therefore, the objective of this study is to assess the oncologic outcome of prostate cancer (PCa) patients with PAFP LN metastasis in a larger patient population.

**Methods:**

Data were analyzed on 8800 patients from eleven international centers in three countries. Eighty-eight patients were found to have metastatic disease to the PAFP LNs (PAFP+) and 206 men had isolated metastasis to the pelvic LNs (PLN+). Clinicopathologic features were compared using ANOVA and Chi square tests. The Kaplan-Meier method was used to calculate the time to biochemical recurrence (BCR).

**Results:**

Of the eighty-eight patients with PAFP LN metastasis, sixty-three (71.6 %) were up-staged based on the pathologic analysis of PAFP and eight (9.1 %) had a low-risk disease. Patients with LNs present in the PAFP had a higher incidence of biopsy Gleason score (GS) 8–10, pathologic N1 disease, and positive surgical margin in prostatectomy specimens than those with no LNs detected in the PAFP. Men who were PAFP+ with or without PLN involvement had more aggressive pathologic features than those with PLN disease only. However, there was no significant difference in BCR-free survival regardless of adjuvant therapy. In 300 patients who underwent PAFP LN mapping, 65 LNs were detected. It was also found that 44 out of 65 (67.7 %) nodes were located in the middle portion of the PAFP.

**Conclusions:**

There was no significant difference in the rate of BCR between the PAFP LN+ and PLN+ groups. The PAFP likely represents a landing zone that is different from the PLNs for PCa metastasis. Therefore, the removal and pathologic analysis of PAFP should be adopted as a standard procedure in all patients undergoing radical prostatectomy.

**Electronic supplementary material:**

The online version of this article (doi:10.1186/s12894-015-0070-1) contains supplementary material, which is available to authorized users.

## Background

In men undergoing radical prostatectomy (RP), pelvic lymph node dissection (PLND) is the most accurate and reliable staging procedure for detecting lymph node (LN) metastasis in prostate cancer (PCa) [1–4]. Aside from providing clinicians with the most accurate LN staging, the therapeutic role of PLND in PCa has emerged as some have suggested that RP and removal of involved regional LNs has survival benefit [5–7]. Currently, an extended template for PLND has been accepted by many surgeons as the standard due to higher LN yield, increased removal of positive nodes, and fewer missed positive nodes [8, 9]. Nevertheless, the optimal extent of PLND that balances potential morbidity with therapeutic benefit remains controversial.

During RP, Ahlering et al. have proposed that the prostatic anterior fat pad (PAFP) should be dissected to aid in the identification of the puboprostatic ligaments and the anterior surface of the dorsal vein complex [10]. In addition, the potential oncologic rationale for the PAFP removal has been suggested initially by Kothari et al. in 2001 [11]. Since then, several groups have reported the presence of LNs in the PAFP and incidence of PAFP LN metastasis occurring in the range of 5.5 % to 17.0 % and 1.2 % to 2.5 %, respectively [12–16].

Most recently, we have reported the largest series on the pathologic analysis of LNs and LN metastasis in the PAFP after reviewing 4,261 patients from 8 institutions [17]. In this study, PAFP LNs were found in 11.9 % and 0.94 % harbored metastatic disease. More importantly, our initial study suggested that metastatic disease to the PAFP LN was associated with more favorable oncologic outcome than those that involve the PLNs. To further define the oncologic implications of PAFP LN metastasis, we have expanded the scope of the study to 8800 men from 11 international institutions.

## Methods

### Ethics statement

This study was approved by the institutional review board of all 13 participating institutions (see Additional file 1). Furthermore, the principles of the Helisinki Declaration were followed. Each board exempted informed consent because this was a retrospective study.

### Study population

Prospectively maintained database approved by the institutional review board (IRB) at each institution was analyzed. Written informed consent was obtained from all study subjects during the study period between January of 2006 and February of 2014. In this study, only men who underwent PAFP excision and pathologic analysis during open retropubic radical prostatectomy (RRP) or robot-assisted radical prostatectomy (RARP) were included. All patients routinely undergo PAFP excision and pathologic analysis from the thirteen participating institutions. Initially, the outcomes of 9510 PCa patients were reviewed [RARP, *N* = 8747 and RRP, *N* = 763]. Of these, 8800 PCa patients from eleven institutions with complete data were selected for analysis.

The participating thirteen institutions are as follows: Rutgers Cancer Institute of New Jersey (New Brunswick, NJ, USA), University of Pennsylvania (Philadelphia, PA, USA), Yonsei University (Seoul, Korea), University of California Irvine (Orange, CA, USA), Asan Medical Center (Seoul, Korea), Samsung Medical Center (Seoul, Korea), Taichung Veterans General Hospital (Taichung, Taiwan), Temple University (Philadelphia, PA, USA), Associated Medical Professionals (Syracuse, NY, USA), Icahn school of Medicine at Mount Sinai Hospital (New York, NY, USA), City of Hope National Medical Center (Duarte, CA, USA), Kyungpook National University Medical Center (Daegu, Korea), and Georgetown University (Washington, D.C., USA).

### PAFP removal and pathologic evaluation

PAFP removal and pathological analysis were performed as described previously [17].

### Statistical analysis

For comparison of variables, a student *t* test or analysis of variance (ANOVA) test and Pearson χ^2^ test were used for analysis of each set of continuous and categorical data. Biochemical recurrence (BCR) was defined as 2 consecutive PSA increases with the last PSA 0.2 ng/ml or greater. Multivariate Cox regression analyses were performed to identify factors predictive of BCR. The time to BCR was used as the end point for the Kaplan-Meier model. The log-rank test was used for comparison with *p* ≤ 0.05 considered statistically significant. All statistical analyses were performed using the SPSS v.18.0 (IBM Corp., Armonk, NY).

## Results

From the eleven international institutions, 8800 patients underwent pathologic analysis of the PAFP (data not shown; see Additional file 2) because the number of patients with LNs present in the PAFP was not available due to an institutional procedure on not reporting negative LNs at two sites. Metastatic disease in the PAFP was detected in eighty-eight patients out of 8800 (0.93 %). The overall incidence of LNs present in the PAFP was 10.3 % (909/8800).

2835 out of 5260 (53.9 %) patients with available data on pelvic LNs underwent pelvic LN dissection, with varying institutional range from 23.2 % to 100.0 %. For these patients, the mean (median) number of dissected total pelvic LN was 6.1 (7) with values in the range of 1–45. Of the ones who underwent pelvic LN dissection, 4.4 % of patients had metastasis to pelvic LN.

Pre- and post-operative patient characteristics of 8800 men with known LN status in the PAFP is known are summarized in Table 1 with a median follow-up of 18.0 months (range 3.0-84.0 months). In this cohort, 7891 patients were found to have no LNs in the PAFP. Preoperatively, biopsy Gleason score (GS) was the only variable significantly different between the two groups. Specifically, patients with LNs present in the PAFP had more frequent biopsy GS of 8–10 than those with LNs absent in the PAFP. Regarding pathologic characteristics of the RP specimens, statistically significant differences were found for pathologic LN (N) stage (*P* = 0.001) and surgical margin status (*P* < 0.001).

**Table 1:** Pre- and post-operative characteristics of patients with absence or presence of lymph nodes in PAFP

	LN absent in PAFP	LN present in PAFP	P-value
	(N = 7891)	(N = 909)	
Age, years: mean (SD)	62.7 (7.5)	62.9 (7.7)	0.413
BMI, kg/m^2^ :mean (SD)	27.9 (3.7)	27.9 (4.2)	0.814
PSA, ng/ml: mean (SD)	8.84 (12.32)	10.00 (24.62)	0.107
Categorical PSA, ng/ml: %			0.814
0-3.9	20.0	22.0	
4-9.9	58.3	56.2	
10-20	15.1	14.0	
>20	6.7	7.8	
Biopsy GS: %			<0.001
6-7	82.8	78.3	
8-10	17.2	21.7	
Pathologic GS: %			0.307
6-7	83.8	82.1	
8-10	16.7	17.9	
Pathologic T stage: %			0.659
T2≥	67.5	66.8	
T3≤	32.5	33.2	
Pathologic N stage: %			0.001
N0/Nx	96.0	93.5	
N1	4.0	6.5	
Margin status: %			<0.001
Negative	82.3	78.1	
Positive	17.7	21.9	

PAFP, Prostate anterior fat pad; LN, Lymph node; BMI, Body mass index; PSA, Prostate-specific antigen; GS, Gleason score

Table 2 lists the clinicopathologic results of the patients with metastatic disease to the LNs stratified by location. Group 1 had isolated metastasis to the pelvic LNs (PLNs) (n = 206). Group 2 had metastatic disease limited to the PAFP LNs (n = 63). Group 3 involved disease both in the pelvic and PAFP LNs (n = 25). Among the eighty-eight patients with metastasis to the PAFP LNs, eight (9.1 %) had low-risk disease based on the D’Amico criteria and sixty-three (71.6 %, Group 2) were up-staged as a result of the PAFP pathologic analysis. Compared to men with pelvic LNs metastasis only (group 1), patients with metastatic disease to the PAFP LNs (Group 2 and 3) had more aggressive features in biopsy and pathologic GS as well as pathologic stage.

**Table 2:** Differences in clinicopathologic results among the 3 groups stratified by the location of positive lymph nodes.

	Group 1, *N* = 206	Group 2, *N* = 63	Group 3, *N* = 25	*P-value*
Age, years: mean (SD)	63.3 (6.9)	63.3 (7.4)	64.4 (8.1)	0.744
PSA, ng/ml: mean (SD)	21.6 (36.0)	26.9 (85.5)	37.3 (66.5)	0.336
BCR-free survival, months: mean (range)	19.2 (0.7-77.7)	21.6 (1.0-76.3)	19.6 (2.6-60.0)	0.163
BCR: N (%)				0.073
No	145 (70.4)	35 (55.6)	15 (60.0)	
Yes	61 (29.6)	28 (44.4)	10 (40.0)	
D’Amico risk: *N* (%)				0.009
Low risk	29 (14.1)	7 (11.1)	1 (4.0)	
Intermediate risk	65 (31.6)	17 (27.0)	1 (4.0)	
High risk	112 (54.4)	39 (61.9)	23 (92.0)	
Biopsy GS: *N* (%)				<0.001
6-7	116 (56.5)	25 (41.0)	5 (20.0)	
8-10	89 (43.4)	36 (59.0)	20 (80.0)	
Pathologic GS: *N* (%)				0.021
6-7	113 (54.9)	29 (46.0)	8 (32.0)	
8-10	93 (45.1)	34 (54.0)	17 (68.0)	
Pathologic T stage: *N* (%)				0.005
T2	81 (39.3)	18 (28.6)	3 (12.0)	
T3a	48 (23.3)	22 (34.9)	6 (24.0)	
T3b	59 (28.6)	19 (30.2)	10 (40.0)	
T4	18 (8.7)	4 (6.3)	6 (24.0)	
Margin status: *N* (%)				0.043
Negative	69 (33.5)	37 (58.7)	9 (36.0)	
Positive	137 (66.5)	26 (41.3)	16 (64.0)	
Adjuvant therapy: *N* (%)				0.012
No	165 (80.1)	42 (66.7)	16 (64.0)	
Yes	41 (19.9)	21 (33.3)	9 (36.0)	

Group 1, Pelvic LN metastasis only; Group 2, PAFP LN metastasis only; Group 3, Both pelvic LN & PAFP LN metastasis; PSA, Prostate-specific antigen; BCR, Biochemical recurrence; GS, Gleason score

Adjuvant therapy, including androgen deprivation (ADT), radiation, and chemotherapy was performed more frequently in men with PAFP LN involvement. 63 out of 71 patients (88.7 %) who were given adjuvant therapy received ADT with or without radiation and chemotherapy. The remaining 8 patients (11.3 %) did not receive ADT and received radiation, chemotherapy, or both. The median BCR-free survival period for PLN+, PAFP+, and PAFP+/PLN+ were 19.2, 21.6, and 19.6 months, respectively. Currently, fifty patients with PAFP LN metastasis remain free of BCR.

In order to check whether PAFP LN+ was a surrogate for extracapsular extension (ECE+), survival analysis of those with simultaneous ECE+ and PAFP LN+ was compared with that of individuals with ECE- or PAFP LN-. Although the relative frequency of BCR seemed different, the Kaplan-Meier analysis revealed no differences between the two groups: 46.3 % of ECE+/PAFP LN+ group had BCR with the median BCR free survival time of 18.0 months. On the other hand, 30.0 % of ECE- or PAFP- group had BCR with the median BCR free survival time of 15.4 months (P = 0.287). To determine the anatomic location of the LNs with in PAFP, LN mapping was carried out at one institution as reported previously [17]. From the cohort of 300 men, the total number of LNs detected was 65 (Table 3). Of these, 44 (67.7 %) were located in the middle packet. The numbers of LNs found in the left and right segments were 11 (16.9 %) and 10 (15.4 %), respectively.

**Table 3:** Location of lymph nodes within the PAFP

Total # Patients	300
Number of Nodes Detected	657
Middle (# of Nodes)	44
Left (# of Nodes)	11
Right (# of Nodes)	10

The Multivariate Cox regression model suggested that higher preoperative PSA was predictive of higher recurrence rates in all patients (HR 1.005; 95 % CI 1.000-1.009; P = 0.042) and in the subgroup of patients with adjuvant therapy (HR 1.009; 95 % CI 1.001-1.016; P = 0.019). In addition, PLN+, PAFP LN+, and PLN+/PAFP LN+ demonstrated comparable risks of developing BCR (Table 4).

**Table 4:** Multivariate Cox regression analyses to identify predictors of biochemical recurrence

Variables	HR	95 % CI		*P-value*
		Lower	Upper	
All patients				
Age	1.008	.979	1.038	.604
Preoperative PSA	1.005	1.000	1.009	.042
Post-operative GS (≤7 *vs.* ≥8)	1.264	.822	1.943	.286
Pathologic stage (T2 *vs.* T3)	.931	.584	1.484	.763
Margin status (Negative *vs.* Positive)	1.159	.745	1.803	.514
Pelvic and PAFP LN metastasis status				
Group 1	1	-	-	-
Group 2	1.335	.821	2.169	.244
Group 3	1.288	.639	2.594	.479
Patients without adjuvant therapy				
Age	1.020	.985	1.055	.265
Preoperative PSA	1.003	.996	1.009	.423
Post-operative GS (≤7 *vs.* ≥8)	1.094	.648	1.848	.737
Pathologic stage (T2 *vs.* T3)	1.024	.601	1.746	.930
Margin status (Negative *vs.* Positive)	1.094	.648	1.848	.555
Pelvic and PAFP LN metastasis status				
Group 1	1	-	-	-
Group 2	1.350	.754	2.418	.312
Group 3	1.064	.406	2.792	.899
Patients with adjuvant therapy				
Age	.994	.933	1.059	.842
Preoperative PSA	1.009	1.001	1.016	.019
Post-operative GS (≤7 *vs.* ≥8)	1.925	.774	4.788	.159
Pathologic stage (T2 *vs.* T3)	.727	.250	2.117	.559
Margin status (Negative *vs.* Positive)	1.316	.467	3.711	.604
Pelvic and PAFP LN metastasis status				
Group 1	1	-	-	-
Group 2	1.633	.600	4.446	.337
Group 3	2.592	.857	7.842	.092

HR, hazard ratio; CI, confidence interval; Group 1, Pelvic LN metastasis only; Group 2, PAFP LN metastasis only; Group 3, Both pelvic LN & PAFP LN metastasis

**Fig. 1:**
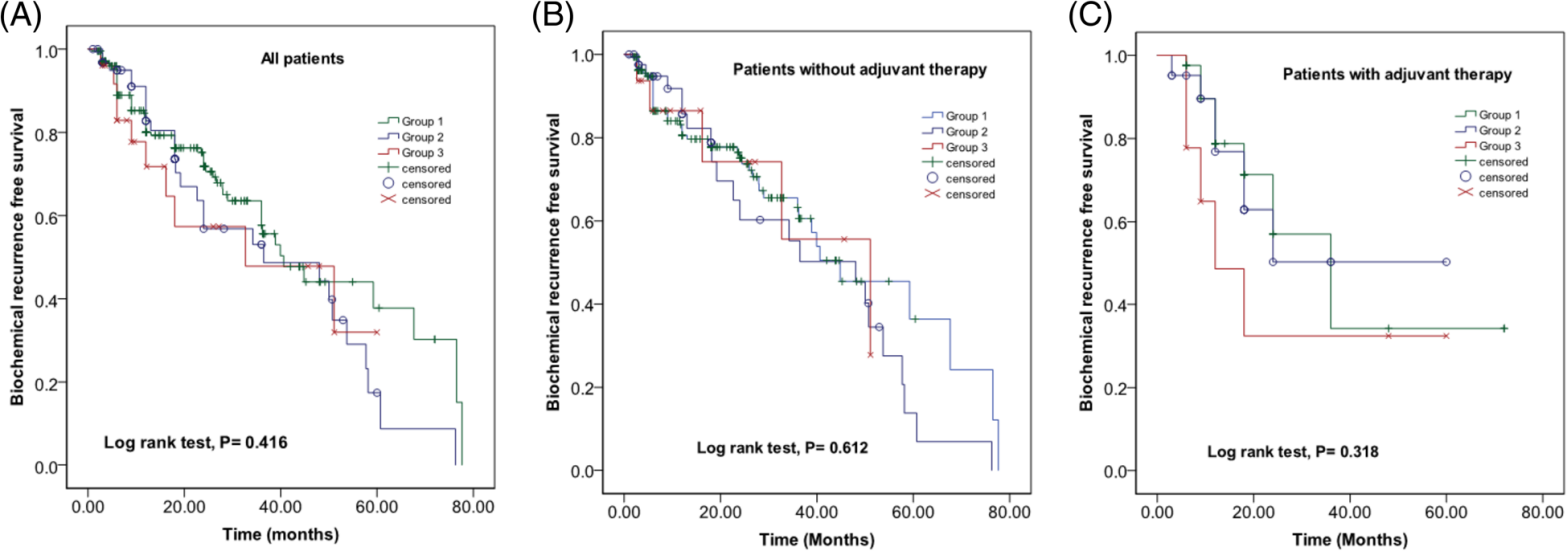
Kaplan-Meier curves for BCR-free survival according to the location of metastatic lymph nodes in (**a**) all patients (**b**) patients without adjuvant therapy, and (**c**) patients with adjuvant therapy

Kaplan-Meier curves were used to assess BCR according to the location of the metastatic LNs (Fig. 1). No statistically significant difference was found in the BCR when all three groups were compared (Fig. 1a). When stratified by the administration of adjuvant therapy, again no difference was observed among the three groups (Fig. 1b and c).

## Discussion

Our international study spanning multiple institutions has demonstrated that in 8800 patients who underwent RP, the overall incidence of metastasis in the PAFP LNs was 0.93 %. Simultaneously, the rate of LNs detected within the PAFP was 10.3 %. LN mapping within the PAFP demonstrated that 67.7 % of the LNs were located in the middle packet. Of the 88 patients with PAFP LN metastasis, 63 were upstaged as a result of the PAFP pathologic evaluation.

When clinicopathologic features were analyzed between men with and without LN in the PAFP, patients with LNs in the PAFP more frequently had biopsy GS 8–10, N1 disease pathologically, and positive surgical margins. In comparison to the patients with isolated metastasis to the pelvic LNs, men with PAFP LNs metastasis had worse pathologic features. Yet, there was no significant difference in BCR free survival when the data were assessed based on the location of the metastasis (pelvic LN+, PAFP LN+, or pelvic LN+/PAFP LN+). Collectively, these observations suggest that the PAFP should be removed in all patients undergoing RP and that the oncologic implication of PAFP LN metastasis is equivalent to that of pelvic LN involvement in men with PCa.

Previously, our group reported on the detailed analysis of 40 patients with metastatic PCa to the PAFP LNs [17]. Because this original report was largely focused on the clinicopathologic features of men with PAFP LN metastasis, we designed the current study to assess the oncologic implications of PAFP LN involvement in men with PCa. To this end, we have increased the sample size to 9510 by increasing the number of participating institutions to thirteen. The study sites represent fourteen urologic surgeons from three different countries – USA, South Korea, and Taiwan.

A careful analysis of 8800 men after excluding patients with incomplete data revealed that men with LNs present in the PAFP were more likely to have aggressive disease as indicated by the higher frequency of biopsy GS 8–10 PCa, pathologic stage N1, and positive surgical margin. In a significantly smaller sample size of 356 men, Hansen et al. similarly reported that pathologic N1 disease was more frequently detected in patients who harbor LNs within the PAFP than those without LNs within the PAFP (21.1 % *vs.* 7 %, *P* = 0.02) [14]. In addition, it has been suggested that patients with LNs found in the PAFP were younger (60.5 *vs. *65.0, *P* = 0.002) [13] while Jeong et al. noted that the mean preoperative PSA level was significantly higher in patients with LNs present in the PAFP (7.70 *vs.* 6.01, *P* = 0.039) [15]. But in the present study, age and PSA did not show any differences between the two groups.

Clinically, the current study demonstrated that the outcome of men with metastatic PCa to the PAFP LNs is similar to that of patients with pelvic LN metastasis. To assess the oncologic significance of PAFP LN metastasis in men with PCa, we have compared the outcome based on the location of the positive LNs (pelvic LN only, PAFP LN only, and pelvic LN+/PAFP LN+) in both Cox regression model as well as Kaplan-Meier survival analysis. Pathologic analysis revealed that men with PAFP LN involvement, regardless of the pelvic LN status, had more aggressive features. Nevertheless, BCR free survival duration was not significantly different among the three groups. More importantly, this lack of difference in BCR free survival period was present regardless of adjuvant therapy (P = 0.469). Moreover, among 88 patients with PAFP LN+, there were 67 patients who had simultaneous ECE+ and PAFP LN+, illustrating a high level of correlation. The risk of BCR in the above group was highly elevated although no statistical difference was found when compared to those with ECE- or PAFP-: (31/67) 46.3 % vs. (68/227) 30.0 %, respectively (P = 0.287). Taken together, these findings suggest that PCa patients with metastasis to the PAFP LNs should be treated as those with pelvic LN metastasis.

Finally, results of the present study provide multiple reasons for the PAFP removal and pathologic analysis in all men undergoing RP. First, the PAFP LNs are likely an independent and separate anatomic landing zone for PCa metastasis. In our group’s initial publication, we have reported that the LNs within the PAFP overwhelmingly mapped to the middle packet [17]. In this update, we have increased the sample size and carried out LN mapping in 300 patients. Again, a significant majority (67.7 %) of the LNs in PAFP were located in the middle packet. Accordingly, the detection of LNs within PAFP is not likely a result of an incomplete dissection of the obturator LNs. Second, the pathologic analysis of PAFP enhances the accuracy of staging. Of the 88 men with metastatic disease to the PAFP LNs, 63 were upstaged based on the PAFP LNs involvement. Third, there are no reliable pre-operative parameters that predict the PAFP LN metastasis. Although no preoperative imaging is currently recommended for the detection of PAFP LN metastasis and for guidance in removing PAFP LN, the added surgical step in the absence of imaging modality will not likely compromise the quality of surgical outcomes. In our aforementioned initial multi-institution study that analyzed forty patients with PAFP LN disease, only three had a low-risk disease defined by the D’Amico criteria pre-operatively. Based on this observation, we suggested that the pathologic analysis of PAFP may not be necessary in men with low-risk PCa. However in the current study, 9.1 % had low-risk disease. Accordingly, all PAFP specimens should be analyzed pathologically. Fourth, there may be a therapeutic effect of PAFP removal. Of the 88 men with PAFP LN metastasis, fifty remain free of BCR. Taken together with the minimal surgical morbidity of PAFP dissection, we now contend that the removal and pathologic examination be a standard procedure in all patients undergoing RP.

Notwithstanding the strength of the largest sample size to date on this topic, our study is not without weaknesses. First, the number of men with PAFP LN metastasis was only 88. Given this small number of event, it is entirely possible that there is a unique oncologic implication of PAFP LN metastasis that requires a larger sample size to uncover. Indeed in this cohort, PAFP LN involvement, regardless of the pelvic LN status, had more aggressive pathologic features. Second, additional follow-up is necessary to evaluate cancer-specific and overall survival. Third, BCR comparisons among pelvic LN+, PAFP LN+, and PAFP LN+/Pelvic LN+ groups were likely confounded because a greater proportion of men with PAFP LN+ with and without pelvic LN+ (group 2 and 3) received adjuvant therapy than the men with pelvic LN+ only (group 1) (P = 0.012) (Table 2). Because adjuvant therapy may lower BCR, adjuvant therapy-adjusted BCR in group 2 and 3, may in fact, be higher. Hence, this finding may further support the substantial BCR risk associated with PAFP metastasis. We plan to continue increasing the overall sample size and track the patients with PAFP LN metastasis to determine the long-term oncologic outcome. In the meantime, the present study provides the relative confidence that PCa patients with PAFP LN metastasis should be treated as those with pelvic LN disease.

## Conclusions

Metastasis to the PAFP LNs and pelvic LNs had equivalent duration of BCR free survival. Because the PAFP is likely an anatomically independent and separate landing zone of PCa metastasis, the PAFP should be removed and analyzed in all men undergoing RP.

## Additional files

Additional file 1:
**Ethics statement. **docx. (DOCX 81 kb) Additional file 2:
**Institutional breakdown** **(Table S1).** docx (DOCX 17 kb)

## Abbreviations

BCR: Biochemical recurrence
ECE: Extracapsular extension
GS: Gleason score
LN: Lymph node
PAFP: Prostatic anterior fat pad
PCa: Prostate cancer
PSA: Prostate-specific antigen
PAFP+: Metastasis to prostatic anterior fat pad lymph node
PNL+: Metastasis to pelvic lymph node
PLN: Pelvic lymph node
RARP: Robot-assisted radical prostatectomy
RRP: Retropubic radical prostatectomy
