# Supplementary material for: Oncologic outcomes in men with metastasis to the prostatic anterior fat pad lymph nodes: a multi-institution international study
Source: BMC Urol. 2015 Aug 1;15:79. doi: 10.1186/s12894-015-0070-1 (PMC4521494; doi:10.1186/s12894-015-0070-1)
Supplement: Additional file 1: — Ethics statement. docx. (DOCX 81 kb) [file 12894_2015_70_MOESM1_ESM.docx]

***Ethics statement***

This study was approved by the institutional review board of all 13 participating institutions listed below:

1. Rutgers Cancer Institute of New Jersey and Rutgers Robert Wood Johnson Medical School, New Brunswick, NJ, USA
2. Kyungpook National University Medical Center, Daegu, Korea
3. University of Pennsylvania, Philadelphia, PA, USA
4. Yonsei University, Seoul, Korea
5. UC Irvine School of Medicine, Orange, CA, USA
6. Asan Medical Center, University of Ulsan College of Medicine, Seoul, Korea
7. Samsung Medical Center, Sungkyunkwan University School of Medicine, Seoul, Korea
8. Taichung Veterans General Hospital, Taichung, Taiwan
9. Temple University School of Medicine, Philadelphia, PA, USA
10. Associated Medical Professionals, Syracuse, NY, USA
11. Icahn school of Medicine at Mount Sinai Hospital, New York, NY, USA
12. City of Hope National Medical Center, Duarte, CA, USA
13. Georgetown University, Washington, DC, USA
