# Supplementary material for: Oncologic outcomes in men with metastasis to the prostatic anterior fat pad lymph nodes: a multi-institution international study
Source: BMC Urol. 2015 Aug 1;15:79. doi: 10.1186/s12894-015-0070-1 (PMC4521494; doi:10.1186/s12894-015-0070-1)
Supplement: Additional file 2: — Institutional breakdown (Table S1). docx (DOCX 17 kb) [file 12894_2015_70_MOESM2_ESM.docx]

Supplementary Table 1. Institutional breakdown of PAFP lymph node presence and metastasis.

| Institution Number | Sample size | PAFP LN absent | PAFP LN present | PAFP LN metastasis |
| --- | --- | --- | --- | --- |
| 1 | 817 | 756 | 61 | 11 |
| 2 | 340 | 268 | 72 | 5 |
| 3 | 464 | 443 | 21 | 3 |
| 4 | 182 | * | * | 2 |
| 5 | 151 | 139 | 12 | 1 |
| 6 | 2141 | 1948 | 193 | 9 |
| 7 | 991 | 854 | 137 | 8 |
| 8 | 798 | 765 | 33 | 4 |
| 9 | 610 | 567 | 43 | 5 |
| 10 | 549 | 469 | 80 | 6 |
| 11 | 903 | 761 | 142 | 16 |
| 12 | 1036 | 921 | 115 | 15 |
| 13 | 528 | * | * | 3 |
| Total | 9510 | 7891 | 909 | 88 |
